# Increases in regimen durability associated with the introduction of tenofovir at a large public-sector clinic in Johannesburg, South Africa

**DOI:** 10.7448/IAS.16.1.18794

**Published:** 2013-11-19

**Authors:** Alana T Brennan, Mhairi Maskew, Prudence Ive, Kate Shearer, Lawrence Long, Ian Sanne, Matthew P Fox

**Affiliations:** 1 Center for Global Health & Development, Boston University, Boston, MA, USA; 2 Health Economics and Epidemiology Research Office, Faculty of Health Sciences, Department of Internal Medicine, School of Clinical Medicine, University of the Witwatersrand, Johannesburg, South Africa; 3 Clinical HIV Research Unit, Faculty of Health Sciences, Department of Internal Medicine, School of Clinical Medicine, University of the Witwatersrand, Johannesburg, South Africa; 4 Right to Care, Johannesburg, South Africa; 5 Department of Epidemiology, Boston University School of Public Health, Boston, MA, USA

**Keywords:** antiretroviral therapy, single-drug substitution, resource-limited setting, drug toxicities, drug side effects, regimen durability

## Abstract

**Introduction:**

In April 2010, tenofovir replaced stavudine in public-sector first-line antiretroviral therapy (ART) in South Africa. The association of tenofovir with fewer side effects and toxicities compared to stavudine could translate to increased durability of tenofovir-based regimens. We evaluated changes over time in regimen durability at the Themba Lethu Clinic, Johannesburg, South Africa.

**Methods:**

This was a cohort analysis of treatment-naïve, non-pregnant adult patients initiated on ART between April 2004 and December 2011. First-line ART regimens before April 2010 consisted of stavudine or zidovudine with lamivudine and either efavirenz or nevirapine. Tenofovir was substituted for stavudine after April 2010. We evaluated the frequency and type of single-drug substitutions (excluding switches to second-line therapy). Cox models were used to evaluate the association of ART initiation year and antiretroviral drug type with single-drug substitutions in the first 12 months on treatment.

**Results:**

One thousand nine hundred and sixty-four (10%) substitutions occurred amongst 19,699 patients. Excluding 2004 (year of treatment roll-out), before 2010 one-year single-drug substitutions ranged from 10.0 to 13.1%. In 2011, well after integration of tenofovir, substitutions decreased to 5.6%. Single-drug substitution was lowest amongst patients on tenofovir (5.1%) versus zidovudine (11.3%), 30 mg stavudine (10.5%) or 40 mg stavudine (14.4%). Adjusted Cox models showed that patients initiating treatment between 2005 and 2010 (vs. 2011) had a twofold increased hazard of single-drug substitution, while those on zidovudine or stavudine had a two to threefold increase in single-drug substitution versus tenofovir patients in the first 12 months on ART.

**Conclusions:**

The decline in single-drug substitutions is associated with the introduction of tenofovir. Tenofovir use could improve regimen durability and treatment outcomes in resource-limited settings.

## Introduction

The success of scale-up of antiretroviral therapy (ART) in resource-limited settings like South Africa is in large part due to the introduction of a public health approach to access treatment advocated by the World Health Organization (WHO) that emphasized standardized treatment regimens that could be purchased in large quantities and delivered at scale [1, 2]. Currently, South Africa's national treatment guidelines allow for the use of seven antiretroviral drugs (ARVs) for first- and second-line therapies [3]. As treatment options are limited, increasing regimen durability by minimizing the rates of substituting individual drugs within first-line therapy (hereafter referred to as “single-drug substitutions”) and rates of treatment failure amongst those on first-line regimens is essential to extend first-line treatment options and prevent premature initiation of second-line therapy.

Prior to the South African National Guidelines change in 2010, the most frequently used nucleoside reverse-transcriptase inhibitor (NRTI), with the exception of lamivudine, was stavudine [2]. Stavudine is effective [4, 5] but is associated with severe side effects, such as dyslipidaemias, lipoatrophy and mitochondrial toxicities, notably peripheral neuropathy and lactic acidosis [6–10]. In 2007, as a result of stavudine's poor side-effect profile, the WHO recommended reducing standard stavudine dosage from 40 mg to 30 mg for all adults on ART [11], and in 2009 recommended discontinuing stavudine for initial HIV treatment [1]. Currently, the WHO recommends giving preference to the use of tenofovir and efavirenz in standard first-line therapy when possible [12]. In 2010, the South African government followed this advice and substituted stavudine with tenofovir, a nucleotide analogue reverse-transcriptase inhibitor (NtRTI), for all new ART initiates [3].

In resource-limited settings, the transition from stavudine to tenofovir has been slow due to cost [13] and management of toxicities, principally renal insufficiency [12], which occurs in about 3% of the population of people living with HIV (PLHIV) [14–20]. By 2011, 84 out of 87 reporting countries had adopted international guidelines that recommend shifting away from stavudine-based to zidovudine- or tenofovir-based first-line regimens [21]. Although many countries have adopted the 2010 WHO guidelines, the phase-out of stavudine remains slow. In South Africa, there has been a substantial decrease in the number of patients on stavudine, from 67% in 2006 to 30% in 2013, with an estimated 715,300 adults still on a stavudine-based regimen [22].

To date, four observational studies set in resource-limited settings have compared single-drug substitutions amongst patients on tenofovir-, stavudine- (30 mg) and zidovudine-based regimens using routine clinical programmme data. All four studies found that those on zidovudine and stavudine were at increased risk of single-drug substitution compared to patients on tenofovir [23–26]. In addition, results of a recent study conducted in a resource-rich setting showed a substantial decrease in the annual rate of substitutions due to NRTIs, NtRTIs and non-nucleoside reverse-transcriptase inhibitors (NNRTIs) from 20% in 2006 to 5.6% in 2012 amongst PLHIV on treatment; the findings are most likely due to the introduction of new ARVs with better toxicity profiles [27]. Although the study did not look at stavudine, it did show that patients receiving zidovudine had a sevenfold increase in the rate of single-drug substitution compared to those receiving tenofovir. We investigated whether single-drug substitutions have decreased with the introduction of tenofovir into standard first-line therapy in a large government HIV clinic in Johannesburg, South Africa. In addition, because of the continued discussion regarding the potential decrease in associated toxicities with a lower dose of stavudine (40–30 mg), we assessed whether the reduced dose of the drug was associated with fewer single-drug substitutions.

## Methods

### Cohort description

Themba Lethu Clinic, situated at the Helen Joseph Hospital, has enrolled close to 35,000 patients in care, of whom over 25,800 have initiated ART since April 2004. Since the wide-scale roll-out of ART, clinic staff have provided HIV care according to South African National Department of Health guidelines [2, 3], which were updated in 2011 to increase the CD4 cell count eligibility threshold for initiating ART from ≤200 to ≤350 cells/mm^3^ or a WHO stage IV condition [28].

Details of the Themba Lethu clinical cohort have been described previously [29]. Briefly, all data, including demographic, clinical conditions, laboratory test results and medications (ARV and non-ARV related), are entered into TherapyEdge-HIV™ in real time by either a clinician or a data entry clerk at the clinic. Routine laboratory tests (CD4 cell count, full blood counts, liver function tests and renal function tests), with the exception of viral loads, are conducted at the time of ART initiation. Additional testing (tuberculosis microscopy and culture results, lactate levels and glucose and lipid profiles) is performed when clinically indicated. Prior to 2010, CD4 and viral load measurements (taken concurrently with full blood counts, liver function tests and renal function tests) were repeated every six months, but changes to the April 2010 guidelines called for these labs to be measured at 6 and 12 months post-treatment initiation and then yearly thereafter. Although the schedule for clinic visits varies depending on the regimen, typically, patients on treatment are seen for medical follow-up visits and at months 1, 3, 6, 7 or 8, 12 and 13, then every six months thereafter. In regard to ARV pickups, patients have a pharmacy visit at months 1, 3, 6, 7 or 8, 10, 11 and 13, and then every three months thereafter [3].

Adverse event monitoring during the first 12 months on treatment is conducted by clinicians during medical visits in accordance with national guidelines [2, 3]. Typically, alanine aminotransferase levels are routinely measured to monitor liver function for patients on nevirapine; full blood counts are measured at months 1, 2, 3 and 6 to monitor iron levels for those on zidovudine; and creatinine levels are measured at months 3 and 6 for those on tenofovir to monitor renal function [2, 3]. Clinical judgement is used to assess whether additional safety bloods are required if a patient presents with an adverse event. Data on all prescribed ARVs are captured in the database, along with documentation of start and stop dates of all drugs and reasons for discontinuation.

Use of Themba Lethu Clinic data was approved by the Human Research Ethics Committee of the University of the Witwatersrand. Approval for analysis of de-identified data was granted by the Institutional Review Board of Boston University.

### Eligibility criteria

We performed a cohort analysis of data collected prospectively as part of routine care at Themba Lethu Clinic. We included all ART-naïve, non-pregnant PLHIV ≥18 years old who initiated a standard, public-sector, first-line ART regimen at the clinic between April 2004 and December 2011. Prior to April 2010, guidelines called for all male patients, as well as female patients on injectable contraception and using condoms, to be initiated on a regimen of stavudine or zidovudine with lamivudine and efavirenz, while women unable to guarantee reliable contraception were initiated on nevirapine instead of efavirenz [2] due to concerns about the possible association of efavirenz with birth defects [1]. After April 2010, tenofovir replaced stavudine [3]. Until the end of 2007, stavudine dosing was weight based, with Department of Health guidelines calling for 30 mg doses for those weighing less than 60 kg and 40 mg doses for those weighing 60 kg or more [2]. From October 2007, a universal 30 mg dose was introduced, and 40 mg tablets of stavudine were removed from the clinic [2, 3].

### Reasons for single-drug substitutions

Details of possible drug substitutions under treatment guidelines and changes over time are summarized in Table 1. Briefly, before April 2010, if a patient was suffering from side effects and toxicities related to stavudine or zidovudine, and was not in need of second-line therapy for virologic failure, the recommendation was to substitute stavudine with either zidovudine (if no related anaemia or neutropenia was present) or abacavir and to substitute zidovudine with either stavudine or abacavir [2]. After April 2010, patients initiated on stavudine or zidovudine now had a third option, tenofovir, if no signs of renal insufficiency were detected, while those initiated on tenofovir could substitute with stavudine, zidovudine or abacavir [3]. Since 2004, patients initiated on the NNRTI efavirenz could substitute with nevirapine, while those on nevirapine could substitute with efavirenz, except in early pregnancy [2, 3].

**Table 1:** 2004 and 2010 South African national treatment guidelines: recommended substitutions for specific side effects and toxicities for antiretroviral drugs

	Common toxicities	Drug substitution 2004–2010	Drug substitution 2010–the present
Nucleotide analogue reverse-transcriptase inhibitor			
Tenofovir	Renal insufficiency	Tenofovir not available	Stavudine, zidovudine (if no related anaemia or neutropenia) or abacavir
Nucleoside reverse-transcriptase inhibitors			
Stavudine	Neuropathy, hyperlactatemia or lactic acidosis, lipodystrophy or pancreatitis	Zidovudine (if no related anaemia or neutropenia) or abacavir	Tenofovir (if renal function is normal), zidovudine (if no related anaemia or neutropenia) or abacavir
Zidovudine	Anaemia or neutropenia	Stavudine or abacavir	Tenofovir (if renal function is normal), stavudine or abacavir
Non-nucleoside reverse-transcriptase inhibitors			
Efavirenz	Persistent central nervous system toxicity Pregnancy	Nevirapine	Nevirapine
Nevirapine	Hepatotoxicity, severe rash (but not life threatening) or life-threatening rash	Efavirenz (except during early pregnancy)	Efavirenz (except during early pregnancy)

Table adapted from South African national antiretroviral treatment guidelines 2004 [2] and 2010 [3].

### Study variables

The primary outcome variable was the proportion of subjects who underwent a single-drug substitution in the first 12 months on ART amongst patients newly initiated on first-line therapy. For the purposes of this study, a single-drug substitution was defined as changing one drug without initiating a protease inhibitor, and therefore we excluded all switches to second-line therapy. The severity of side effects and toxicities was not assessed. Follow-up time began at ART initiation. For the analyses of single-drug substitution, person-time ended at the earliest of (1) single-drug substitution, (2) initiation on second-line ART, (3) discontinuation of treatment, (3) loss, (4) death, (5) transfer, (6) completion of 12 months of follow-up or (7) closure of the data set (31 December 2012).

### Statistical analysis

Patient baseline characteristics were summarized with descriptive statistics and stratified by year of ART initiation. We calculated the frequency of single-drug substitutions in the first 12 months on treatment from April 2004 to December 2012, stratified by year of ART initiation and type of ARV. Details of which drugs were substituted and the reasons for discontinuation were identified where possible. As tenofovir was only introduced into standard first-line treatment in April 2010, a model containing both year of initiation on ART and type of ARV did not converge. As a result, we ran two separate Cox proportional-hazard regression models to evaluate the association of single-drug substitution in the first 12 months on ART with (1) year of initiation on ART (Model 1) and (2) type of ARV in first-line ART (Model 2). Since one of our main objectives was to assess regimen durability with the introduction of tenofovir, we conducted a subanalysis where we restricted the outcome to NRTI substitutions only (*n*=1610) within standard first-line therapy. We were also interested in examining predictors of single-drug substitution amongst patients initiated on a tenofovir-based regimen only (*n*=4329). In order to do, so we reran Model 2 (with the addition of creatinine clearance levels at ART initiation) and restricted it to only patients initiated on tenofovir after the 2010 change in the guidelines.

Data for CD4 cell count (9.2% missing), haemoglobin (13.6% missing) and body mass index (15.7% missing) at ART initiation were not available for all patients. To account for the missingness, we used multiple imputation by the chained-equations method using the PROC MI command in SAS [30] and assumed that the data were missing at random [31]. All prediction equations included log age at initiation of treatment, gender, square root of CD4 cell count at ART initiation, haemoglobin at ART initiation (continuous), body mass index at ART initiation (continuous), WHO stage (I/II, III and IV) and tuberculosis at ART initiation. Indicator variables for single-drug substitution, death and loss to follow-up were also added to the equations but were not imputed. All models were fitted using 25 imputed data sets, and estimated coefficients were combined by averaging with the MIANALYZE procedure in SAS [32]. Appropriate standard errors were calculated using the within- and between-imputation standard errors of the estimates using Rubin's rules [31].

## Results

In total, 19,699 treatment-naïve, non-pregnant patients ≥18 years of age initiated a standard first-line ART regimen between April 2004 and December 2011. Median time on treatment was 12 months (interquartile range (IQR): 12–12 months). At ART initiation, patients had a median CD4 cell count of 105 cells/mm^3^ (IQR: 40–179), were predominately female (61.9%) and had a median age of 36.6 years (IQR: 31.3–43.2) (Table 2). A total of 1964 (10%; 95% confidence interval (CI): 9.6–10.4%) single-drug substitutions occurred in this cohort over the period of follow-up. Of those 1964 single-drug substitutions, 1610 (82%) substituted an NRTI only and 354 (18%) substituted an NNRTI only in the first 12 months on ART.

**Table 2:** Clinical and demographic characteristics at ART initiation and outcomes at 12 months of follow-up, stratified by year initiated on treatment at Themba Lethu Clinic, Johannesburg, South Africa (*n=*19,699)

Characteristics	2004 (n=1434) n (%)	2005 (n=2065) n (%)	2006 (n=2696) n (%)	2007 (n=2513) n (%)	2008 (n=2250) n (%)	2009 (n=2877) n (%)	2010 (n=2927) n (%)	2011 (n=2937) n (%)	Total (n=19699) n (%)
Regimen base									
Tenofovir*	0 (0.0)	0 (0.0)	0 (0.0)	0 (0.0)	0 (0.0)	0 (0.0)	1765 (60.3)	2564 (87.3)	4329 (22.0)
Zidovudine**	49 (3.4)	101 (4.9)	145 (5.4)	155 (6.2)	197 (8.8)	164 (5.7)	95 (3.3)	109 (3.7)	1015 (5.2)
Stavudine (30 mg)**	683 (47.6)	1140 (55.2)	1634 (60.6)	1819 (72.4)	2012 (89.4)	2707 (94.1)	1066 (36.4)	264 (9.0)	11325 (57.5)
Stavudine (40 mg)**	702 (49.0)	824 (39.9)	917 (34.0)	539 (21.5)	41 (1.8)	6 (0.2)	1 (0.03)	0.0 (0.0)	3030 (15.4)
Non-nucleoside reverse transcriptase inhibitor									
Nevirapine	155 (10.8)	213 (10.3)	289 (10.7)	242 (9.6)	236 (10.5)	294 (10.2)	243 (8.3)	252 (8.6)	1924 (9.8)
Efavirenz	1279 (89.2)	1852 (89.7)	2407 (89.3)	2271 (90.4)	2014 (89.5)	2583 (89.8)	2684 (91.7)	2685 (91.4)	17775 (90.2)
Gender									
Female	971 (67.7)	1345 (65.1)	1639 (60.8)	1556 (61.9)	1407 (62.5)	1706 (59.3)	1850 (63.2)	1722 (58.6)	12196 (61.9)
Male	463 (32.3)	720 (34.9)	1057 (39.2)	957 (38.1)	843 (37.5)	1171 (40.7)	1077 (63.8)	1215 (41.4)	7503 (38.1)
Age (years)									
18–24.9	53 (3.7)	96 (4.7)	147 (5.5)	126 (5.0)	111 (4.9)	109 (3.8)	143 (5.0)	145 (4.9)	930 (4.7)
25–29.9	248 (17.3)	345 (16.7)	398 (14.8)	389 (15.5)	321 (14.3)	409 (14.2)	415 (14.2)	396 (13.5)	2921 (14.8)
30–39.9	683 (47.6)	1008 (48.8)	1276 (47.3)	1188 (47.3)	1012 (45.0)	1238 (42.0)	1250 (42.7)	1175 (40.0)	8830 (44.8)
40–49.9	327 (22.8)	474 (23.0)	646 (24.0)	607 (24.2)	579 (25.7)	797 (27.7)	774 (26.4)	831 (28.3)	5035 (25.6)
≥50	123 (8.6)	142 (6.9)	229 (8.5)	203 (8.1)	227 (10.1)	324 (11.3)	345 (11.8)	390 (13.3)	1983 (10.1)
Age at ART initiation median (IQR)	35.7 (30.9–42.0)	35.4 (30.8–41.5)	36.0 (31.2–42.2)	36.0 (31.0–42.1)	37.0 (31.6–43.5)	37.4 (32.0–44.4)	37.1 (31.5–43.9)	37.8 (31.7–44.8)	36.6 (31.3–43.2)
CD4 at ART initiation									
0–49 cells/mm^3^	494 (34.5)	699 (33.9)	966 (35.8)	836 (33.3)	704 (31.3)	704 (24.5)	682 (23.3)	602 (20.5)	5687 (28.9)
50–99 cells/mm^3^	387 (27.0)	424 (20.5)	555 (20.6)	497 (19.8)	405 (18.0)	535 (18.6)	514 (17.6)	448 (15.3)	3765 (19.1)
100–199 cells/mm^3^	441 (30.8)	710 (34.4)	852 (31.6)	859 (34.2)	804 (35.7)	989 (34.4)	991 (33.9)	952 (31.4)	6598 (33.5)
≥200 cells/mm^3^	112 (7.8)	232 (11.2)	323 (12.0)	321 (12.8)	337 (15.0)	649 (22.6)	740 (25.3)	935 (31.8)	3649 (18.5)
CD4 at ART initiation (cells/mm^3^) median (IQR)	77 (35–138)	89 (34–157)	82.2 (28.5–159)	91 (33–160)	101 (37–171)	119 (59–192)	124 (54.9–201)	146 (64–225)	105 (40–179)
WHO stage at ART initiation									
I/II	836 (58.3)	1097 (53.1)	1399 (51.9)	1498 (95.6)	1374 (61.1)	2011 (69.9)	1979 (67.6)	2165 (73.7)	12359 (62.7)
III	537 (37.5)	852 (41.3)	1063 (39.4)	780 (31.0)	713 (31.7)	681 (23.7)	761 (26.0)	582 (19.8)	5969 (30.3)
IV	61 (4.3)	116 (5.6)	234 (8.7)	235 (9.4)	163 (7.2)	185 (6.4)	187 (6.4)	190 (6.5)	1371 (7.0)
Tuberculosis at ART initiation									
Yes	180 (12.6)	423 (20.5)	511 (19.0)	326 (13.0)	261 (11.6)	330 (11.5)	320 (10.9)	196 (6.7)	2547 (12.9)
No	1254 (87.5)	1642 (79.5)	2185 (81.1)	2187 (87.0)	1989 (88.4)	2547 (88.5)	2607 (89.1)	2741 (93.3)	17152 (87.1)
Haemoglobin at ART initiation (g/dL)									
Median (IQR)	11.7 (10.2–13.1)	11.5 (9.9–13.0)	11.0 (9.9–13.0)	11.6 (9.8–13.1)	12.0 (10.0–13.0)	13.0 (10.0–13.0)	11.6 (10.0–13.1)	11.9 (10.3–13.5)	11.6 (10.1–13.2)
Body mass index at ART initiation (kg/m^2^)									
Median (IQR)	21.3 (19.2–26.1)	21.4 (18.6–25.3)	21.5 (18.7–25.4)	21.6 (18.7–25.7)	21.8 (19.1–25.8)	21.7 (18.9–26.0)	22.6 (19.0–27.7)	22.4 (19.2–27.5)	21.9 (18.9–26.2)
Vital status over 12-months of follow-up									
Death n (%)	99 (6.9)	166 (8.0)	226 (8.4)	220 (8.8)	194 (8.6)	252 (8.8)	181 (6.2)	87 (3.0)	1425 (7.2)
Loss to follow-up n (%)	100 (7.0)	184 (2.9)	266 (9.9)	273 (10.9)	167 (7.4)	324 (11.3)	401 (13.7)	514 (17.5)	2229 (11.3)
Transfers n (%)	35 (2.4)	42 (2.0)	78 (2.9)	106 (4.2)	97 (4.3)	117 (4.1)	183 (6.3)	163 (5.6)	821 (4.2)
Alive n (%)	1200 (83.7)	1673 (81.0)	2126 (78.9)	1914 (76.2)	1792 (79.6)	2184 (75.9)	2162 (73.9)	2173 (74.0)	15224 (77.3)
Primary outcome over 12-months of follow-up									
Single-drug substitution n (%)	103 (7.2)	234 (11.3)	294 (10.9)	330 (13.1)	227 (10.1)	321 (11.2)	290 (9.9)	165 (5.6)	1964 (10.0)
Rates (per 100 pys.)	8.3 (6.8–10.1)	13.7 (12.0–15.5)	13.4 (11.9–15.0)	16.6 (14.9–18.5)	12.6 (11.0–14.3)	14.0 (12.5–15.6)	12.7 (11.3–14.2)	7.1 (6.1–8.2)	12.4 (11.9–13.0)

ART: antiretroviral therapy; IQR: interquartile range; WHO: World Health Organization; pys: person-years.

* Nucleotide analogue reverse-transcriptase inhibitor.

** Nucleoside reverse-transcriptase inhibitor.

Even though the clinic made the shift from stavudine-to tenofovir-based first-line ART in 2010, a total of 706 patients initiated a stavudine-based regimen and 164 patients initiated a zidovudine-based regimen after the change in the national treatment guidelines. According to the 2010 guidelines, patients who have contraindications to tenofovir (renal disease or the use of other nephrotoxic drugs, such as aminoglycosides) should be initiated on a zidovudine-based regimen, while those who have contraindications to tenofovir and zidovudine (renal disease and anaemia or the use of other nephrotoxic drugs) should be initiated on a stavudine-based regimen [3]. Seventy-five percent (*n*=123) of the 164 patients initiated on zidovudine had documented renal insufficiency at ART initiation, while 58% (*n*=410) of the 706 patients initiated on stavudine had renal insufficiency and 204 (29%) had moderate to severe anaemia (<10 µg/dL) at treatment initiation.

### Yearly trends in single-drug substitutions

**Figure 1:**
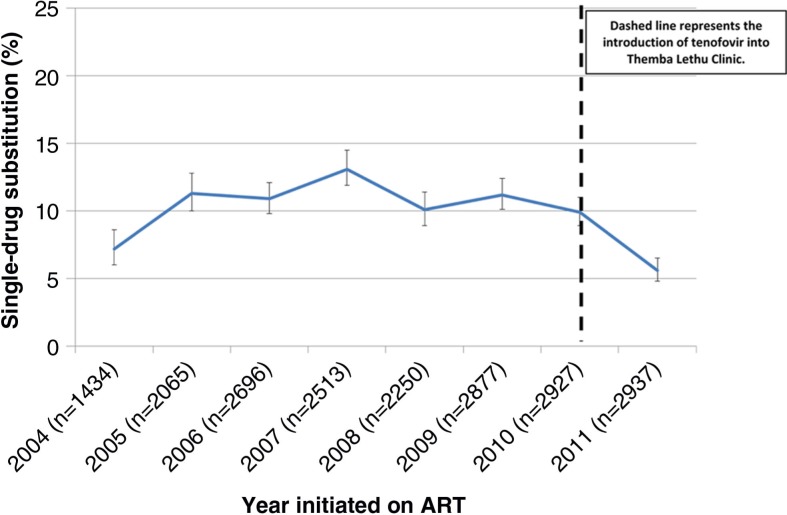
Proportion and 95% confidence interval of single-drug substitutions over the first 12 months on antiretroviral therapy stratified by year of treatment initiation in Johannesburg, South Africa (*N*=19,699). Error bars = 95% confidence intervals.

From 2005 to 2010 (excluding 2004, when treatment roll-out had just begun), prior to the change in the guidelines in 2010, the frequency of single-drug substitutions in the first 12 months on ART ranged from 10.1% (95% CI: 8.9–11.4%) to 13.1% (95% CI: 11.9–14.5%) (Figure 1 and Table 3). In 2011, when the switch to tenofovir was well underway in the clinic (more than 85% of patients were being newly initiated on tenofovir quarterly; see Supplementary file), single-drug substitutions decreased substantially to 5.6% (95% CI: 4.8–6.5%). Results of a Cox proportional-hazard regression model, adjusted for demographic (age and gender) and clinical characteristics at ART initiation (CD4 cell count, haemoglobin, body mass index, tuberculosis and WHO stage), showed that those patients initiated on ART prior to 2011 had approximately a twofold increase in the hazards of single-drug substitution compared to those who initiated treatment during 2011 (Model 1 in Table 3). When restricting the outcome to only NRTI substitutions (*n*=1610), we saw very similar results, albeit slightly increased (Supplementary file).

**Table 3:** Crude and adjusted models for predictors of single-drug substitution in the first 12 months on ART in Johannesburg, South Africa (*n*=19 699)

	Single-drug substitution				
	
Variable	Single-drug substitution events (%)	Person-time (years)	Rate (per 100 person-years) (95% CI)	Crude HR (95% CI)	Adjusted HR (95% CI)
Model 1: year of ART initiation					
Year-initiated ART					
2011	165 (5.6)	2325.4	7.1 (6.1–8.2)	1.0	1.0
2010	290 (9.9)	2283.7	12.7 (11.3–14.2)	1.8 (1.5–2.2)	1.8 (1.5–2.1)
2009	321 (11.2)	2298.0	14.0 (12.5–15.6)	2.0 (1.6–2.4)	2.0 (1.6–2.4)
2008	227 (10.1)	1808.0	12.6 (11.0–14.3)	1.8 (1.5–2.2)	1.7 (1.4–2.1)
2007	330 (13.1)	1986.9	16.6 (14.9–18.5)	2.4 (2.0–2.8)	2.3 (1.9–2.8)
2006	294 (10.9)	2198.0	13.4 (11.9–15.0)	1.9 (1.6–2.3)	1.8 (1.5–2.2)
2005	234 (11.3)	1709.5	13.7 (12.0–15.5)	1.9 (1.6–2.4)	1.8 (1.5–2.3)
2004	103 (7.2)	1234.1	8.3 (6.8–10.1)	1.2 (0.9–1.5)	1.1 (0.9–1.4)
Sex					
Male	610 (8.1)	5904.8	10.3 (9.5–11.2)	1.0	1.0
Female	1354 (11.1)	9938.7	13.6 (12.9–14.4)	1.3 (1.2–1.5)	1.4 (1.2–1.5)
WHO stage at ART initiation					
I/II	1186 (9.6)	10242.7	11.6 (10.9–12.3)	1.0	1.0
III/IV	778 (10.6)	5600.8	13.9 (12.9–14.9)	1.2 (1.1–1.3)	1.2 (1.0–1.3)
CD4 cell count at ART initiation (cells/mm^3^)					
≥ 200	349 (9.6)	3037.2	11.5 (10.3–12.7)	1.0	1.0
100–199	695 (10.5)	5504.2	12.6 (11.7–13.6)	1.1 (1.0–1.2)	1.0 (0.9–1.2)
50–99	380 (10.1)	3038.1	12.5 (11.3–13.8)	1.1 (1.0–1.3)	1.0 (0.9–1.2)
< 50	540 (9.5)	4264.0	12.7 (11.6–13.8)	1.1 (1.0–1.3)	1.0 (0.9–1.2)
Model 2: type of ART					
Regimen base					
Tenofovir*	220 (5.1)	3467.3	6.3 (5.5–7.2)	1.0	1.0
Zidovudine**	115 (11.3)	798.0	14.4 (12.0–17.2)	2.3 (1.8–2.8)	2.8 (2.1–3.7)
Stavudine (30 mg)**	1193 (10.5)	9078.6	13.1 (12.4–13.9)	2.1 (1.8–2.4)	2.7 (2.1–3.3)
Stavudine (40 mg)**	436 (14.4)	2499.7	17.4 (15.9–19.1)	2.8 (2.4–3.3)	3.6 (2.8–4.6)
Non-nucleoside reverse-transcriptase inhibitor					
Efavirenz	1702 (9.6)	14319.7	11.9 (11.3–12.5)	1.0	1.0
Nevirapine	262 (13.6)	1523.8	17.2 (15.2–19.4)	1.4 (1.3–1.7)	1.4 (1.3–1.7)
Sex					
Male	610 (8.1)	5904.8	10.3 (9.5–11.2)	1.0	1.0
Female	1354 (11.1)	9938.7	13.6 (12.9–14.4)	1.3 1.2–1.5)	1.3 (1.2–1.5)
WHO stage at ART initiation					
I/II	1186 (9.6)	10242.7	11.6 (10.9–12.3)	1.0	1.0
III/IV	778 (10.6)	5600.8	13.9 (12.9–14.9)	1.2 (1.1–1.3)	1.2 (1.0–1.3)
CD4 cell count at ART initiation (cells/mm^3^)					
≥ 200	349 (9.6)	3037.2	11.5 (10.3–12.7)	1.0	1.0
100–199	695 (10.5)	5504.2	12.6 (11.7–13.6)	1.1 (1.0–1.2)	1.0 (0.9–1.2)
50–99	380 (10.1)	3038.1	12.5 (11.3–13.8)	1.1 (1.0–1.3)	1.0 (0.8–1.2)
< 50	540 (9.5)	4264.0	12.7 (11.6–13.8)	1.1 (1.0–1.3)	1.0 (0.8–1.1)

CI: confidence interval; HR: hazard ratio; ART: antiretroviral therapy; WHO: World Health Organization.

Models also adjusted for age and additional clinical characteristics at ART initiation (haemoglobin, body mass index and tuberculosis).

* Nucleotide analogue reverse-transcriptase inhibitor.

** Nucleoside reverse-transcriptase inhibitor.

### Nucleotide and nucleoside reverse-transcriptase inhibitors

The frequency of single-drug substitution in the first year on ART treatment was the lowest amongst those patients initiated on tenofovir-based regimens (5.1%; 95% CI: 4.5–5.8%) compared to those initiated on zidovudine- (11.3%; 95% CI: 9.5–13.4%), 30 mg stavudine- (10.5; 95% CI: 10.0–11.1%) or 40 mg stavudine-based regimens (14.4; 95% CI: 13.2–15.7%) (Supplementary file and Table 3). Median time to substitution for patients initiated on tenofovir was 2.0 months (IQR: 0.9–5.6) compared to 3.7 months (IQR: 1.8–6.8) for zidovudine patients, 7.2 months (IQR: 3.7–10.4) for 30 mg stavudine patients and 6.7 months (IQR: 3.9–10.1) for those on 40 mg stavudine. Cox proportional-hazard regression models, adjusted for demographic and clinical characteristics at treatment initiation, showed that those on zidovudine and patients on 30 mg stavudine had a threefold increase in single-drug substitution compared to those on tenofovir over the follow-up period, while those on 40 mg stavudine had close to a fourfold increase (Table 3). When restricting the outcome to those 1610 subjects who substituted NRTI only, results showed that those on zidovudine [adjusted hazard ratio (aHR): 4.5; 95% CI: 3.3–6.0], 30 mg stavudine (aHR: 3.7; 95% CI: 2.9–4.7) and 40 mg stavudine (aHR: 4.9; 95% CI: 3.7–6.4) had even higher hazards of single-drug substitution in the first year of treatment when compared to patients on tenofovir (Supplementary file). When further restricting the model to those patients who initiated ART after the 2010 change in the guidelines and controlling for year of treatment initiation, we found that the impact of calendar year seen in Model 1 (Table 3) was reduced. Patients initiated on ART in 2010 now had a 20% increase in the hazards of single-drug substitution (HR 1.2; 95% CI: 1.0–1.5) compared to those initiated in 2011 (Supplementary file).

### Tenofovir – NtRTI

Cox proportional-hazard regression models, restricted to tenofovir patients who initiated ART after 2010 and adjusted for demographic and clinical characteristics at treatment initiation (*n*=4329), showed that patients with mild [hazard ratio (HR): 1.8; 95% CI: 1.2–2.7], moderate (HR: 3.0; 95% CI: 1.8–5.0) or severe (HR: 2.5; 95% CI: 1.3–4.7) renal insufficiency compared to normal had an increase in the hazards of single-drug substitution in the first 12 months on ART (Table 4). Results also showed that those on nevirapine (vs. efavirenz), those who were older (>50 years vs. 30–40 years) or younger (18–25 years vs. 30–40 years), those initiated on ART in 2010 (vs. 2011) and patients with poorer health status at ART initiation had an increase in the hazards of single-drug substitution over the period of follow-up.

**Table 4:** Crude and adjusted models for predictors of single-drug substitution of tenofovir only in the first 12 months on ART (*n*=4329)

	Single-drug substitution		
	
Variable	Single-drug substitution events (%)	Crude HR (95% CI)	Adjusted HR (95% CI)
Non-nucleoside reverse-transcriptase inhibitor			
Efavirenz	182 (4.5)	1.0	1.0
Nevirapine	38 (12.5)	3.0 (2.1–4.2)	3.3 (2.3–4.8)
Creatinine clearance (mL/min)			
Normal (>90)	125 (4.0)	1.0	1.0
Mild (60–89)	53 (6.4)	1.8 (1.3–2.5)	1.8 (1.2–2.7)
Moderate (30–59)	26 (12.4)	3.3 (2.2–5.0)	3.0 (1.8–5.0)
Severe (<30)	16 (10.5)	2.8 (1.6–4.7)	2.5 (1.3–4.7)
Age at ART initiation (years)			
18–24.9	19 (9.1)	2.1 (1.3–3.4)	1.9 (1.1–3.1)
25–29.9	28 (4.4)	1.0 (0.6–1.5)	0.9 (0.6–1.4)
30–39.9	83 (4.6)	1.0	1.0
40–49.9	52 (4.4)	1.0 (0.7–1.4)	1.0 (0.7–1.4)
≥ 50	38 (7.7)	1.8 (1.2–2.6)	1.7 (1.1–2.6)
Sex			
Male	90 (5.3)	1.0	1.0
Female	130 (4.9)	0.8 (1.7–1.2)	0.8 (0.6–1.1)
Tuberculosis at ART initiation			
No	194 (4.9)	1.0	1.0
Yes	26 (6.9)	1.5 (1.0–2.3)	1.5 (0.9–2.4)
CD4 cell count at ART initiation (cells/mm^3^)			
≥ 200	52 (4.3)	1.0	1.0
100–199	76 (5.2)	1.2 (0.9–1.7)	1.3 (0.8–1.9)
50–99	40 (5.7)	1.4 (0.9–2.1)	1.4 (0.9–2.1)
< 50	52 (5.5)	1.3 (0.9–2.0)	1.3 (0.8–2.0)
Haemoglobin levels at ART initiation			
> 10 µg/dL	158 (4.6)	1.0	1.0
≤ 10 µg/dL	62 (6.7)	1.3 (1.0–1.8)	1.3 (0.9–1.8)
Year of ART initiation			
2011	117 (4.6)	1.0	1.0
2010	103 (5.8)	1.3 (1.0–1.7)	1.3 (1.0–1.7)

HR: hazard ratio; CI: confidence interval; ART, antiretroviral therapy.

Models also adjusted for clinical characteristics at ART initiation (body mass index and WHO stage).

### Non-nucleoside reverse-transcriptase inhibitors

Patients initiated on nevirapine had a higher frequency of single-drug substitution (13.6%; 95% CI: 12.2–15.2%) when compared to those initiated on efavirenz (9.6%; 95% CI: 9.2–10.0%) over the period of follow-up (Table 3). Median time to substitution for those patients initiated on nevirapine was 4.5 months (IQR: 1.4–8.1) compared to 6.4 months for those on efavirenz (IQR: 3.0–10.2). Results of adjusted Cox proportional-hazard regression showed that those on nevirapine had a 40% increase in the hazard of single-drug substitution compared to those on efavirenz (Table 3).

Results from both Models 1 (analysis by year) and 2 (analysis by ARV type) also showed that female versus male patients and those with a WHO stage III/IV versus I/II condition had an increase in the hazards of single-drug substitution in the first 12 months on ART. CD4 cell count at treatment initiation was not a predictor of single-drug substitution.

### Reasons for single-drug substitution

Supplementary file shows the reason for single-drug substitution for the total 1964 substitutions in the cohort, stratified by ARV drug. We were able to obtain the reason for the switch for only 47.5% (*n*=932) of single-drug substitutions. However, for that 47.5%, extensive cleaning was done to determine the exact reason for substitution. Stavudine was substituted predominately due to neuropathy (24.6%), hyperlactataemia or lactic acidosis (15.6%) and lipodystrophy (8.6%). When stratifying stavudine by dose, the frequency of the most common toxicities was lower amongst patients on 30 mg versus 40 mg of stavudine (respectively, neuropathy: 21.5% vs. 33.5%; hyperlactataemia or lactic acidosis: 14.1% vs. 19.6%; and lipodystrophy: 7.2% vs. 12.5%). Zidovudine was substituted due to anaemia (14.5%), lipodystrophy (6.4%) and hyperlactataemia or lactic acidosis (3.6%), and 25.3% of tenofovir substitutions were due to renal insufficiency. In regard to NNRTIs, efavirenz was commonly substituted with nevirapine due to pregnancy (12.8%), while nevirapine was substituted with efavirenz due to tuberculosis treatment (23%) and liver toxicity (18.5%).

## Discussion

With the introduction of tenofovir into standard first-line treatment, it has become clear that the poor side-effect profiles of zidovudine and stavudine have hampered the durability of ART in this setting. As a result of the limited follow-up time since the 2010 change in South African national ART guidelines to include tenofovir in standard first-line therapy, this is one of the first studies to examine changes in single-drug substitutions in this setting in relation to a major policy change. The overall frequency of single-drug substitutions in our cohort was 10% in the first 12 months on ART, which is similar to what has been previously reported [23–27]. We saw a substantial decrease in single-drug substitutions in the first 12 months on ART, from 11.2% in 2009 (prior to the change in South Africa's national ART guidelines) to 5.6% in 2011 (after the replacement of the NRTI stavudine with tenofovir). Since there are fewer side effects and drug toxicities with tenofovir use compared to stavudine [5], it is not surprising that switching to tenofovir in first-line ART would be accompanied by a marked decrease in the number of patients needing single-drug substitutions.

Four previous studies [23–26] conducted in resource-limited settings reported single-drug substitutions occurring more frequently for zidovudine and tenofovir early after treatment initiation and at a higher rate later on in follow-up amongst patients on stavudine, consistent with our results. It is important to note that time to substitution is partly a function of the frequency of monitoring, which differs for each NRTI. Laboratory monitoring for tenofovir and zidovudine is often conducted early on after treatment initiation, while for stavudine monitoring begins more often when the patient begins to develop clinical symptoms of toxicity diagnosed at a medical visit [3]. Our results differed, in regard to the hazards of single-drug substitution, when comparing NRTI drugs. We report a three- to fourfold increase in the hazards of single-drug substitution when comparing stavudine (30 and 40 mg) to tenofovir, while Chi *et al*. (Zambia), Bygrave *et al*. (Lesotho) and Velen *et al*. (South Africa) reported hazards of 1.3, 5.4 and 10.4, respectively [23–25]. Njuguna *et al*. (South Africa) reported hazards of 0.38 and 4.9 for tenofovir and stavudine, respectively, compared to zidovudine [26]. The varying length of follow-up times across studies and varying strategies for monitoring of adverse events may have partly contributed to the difference, as stavudine toxicity increases with time on ART [33]. Median follow-up time in our cohort was 12 months, compared to 7.8, 16.2, 57.4 and 82.4 months in Chi *et al*., Bygrave *et al*., Velen *et al*. and Njuguna *et al*., respectively.

The overall rate of single-drug substitution amongst patients on tenofovir was 6.3 per 100 person-years (95% CI: 5.5–7.2). Results of our proportional-hazard model restricted only to those patients initiated on tenofovir after 2010 showed that patients with renal insufficiency (creatinine clearance <90 mL/min) at ART initiation had a two- to threefold increase in the hazards of single-drug substitution over the period of follow-up, consistent with other studies [24–26]. Twenty-five percent of those who substituted tenofovir for either stavudine or zidovudine did so due to renal insufficiency. Unfortunately, we were unable to determine the reason for substitution in the remaining 75% of patients. However, in these patients, tenofovir was most likely substituted with stavudine or zidovudine due to signs of renal insufficiency or because they were started on nephrotoxic drugs for the treatment of other comorbidities [3]. Regardless, it is imperative that patients on tenofovir are monitored regularly for signs of renal complications. Currently, the national guidelines recommend routine measurement of kidney function in patients at presentation and throughout tenofovir use [3]. At Themba Lethu Clinic, serum creatinine and creatinine clearance are monitored at ART initiation, at three and six months and yearly thereafter for patients on tenofovir [3]. Only patients with a calculated creatinine clearance >50 mL/min can safely start tenofovir [3]. However, if toxicity develops, the clinician has to make the decision to either reduce the dose of tenofovir to every 48 hours, twice weekly or every seven days with haemodialysis or substitute tenofovir with zidovudine [3].

With the slow transition away from stavudine use in resource-limited settings [21, 22], coupled with the fact that patients with contraindications to tenofovir and/or zidovudine only have stavudine as a first-line treatment option, there has been much discussion regarding decreasing the dosage of the drug even lower, to 15 or 20 mg. Although the data on a lower dose of stavudine are scarce, there is evidence that patients can maintain virologic suppression, improve immunologic response and decrease stavudine-related adverse events while on ART [34–37]. One study reported that a switch to a half dose of stavudine (15 or 20 mg) modestly improved mitochondrial function and lessened the loss of bone mineral density [35]. Although our study did not have patients initiated on a stavudine dose less than 30 mg, we did find that patients on 30 mg stavudine had lower hazards of single-drug substitution than those on 40 mg when compared to those on tenofovir, similar to what has been reported previously [38–42].

Our results show that patients on nevirapine had a 40% increase in single-drug substitutions in the first year on treatment compared to patients on efavirenz, consistent with previous findings [43–47]. It is also important to note that female patients in our cohort had a 30% increase in single-drug substitutions in the first year on treatment when compared to men. Some of this increase could be related to pregnancy; however, when we removed women who substituted due to pregnancy, the hazards of single-drug substitution remained unchanged. Nevirapine reactions also have been shown to be more common at higher baseline CD4 cell counts, especially in women with CD4>200 cells/mm^3^ or men with CD4 cell counts>400 cells/mm^3^ [3, 43, 48]. However, when male and female patients who were initiated on nevirapine at higher CD4 cell counts were removed from the analysis, the association between female gender and single-drug substitution also remained unchanged.

Since data for this analysis come from one of the largest HIV clinics in South Africa, which has been actively enrolling patients since the roll-out of treatment in the country in 2004, we were able to evaluate trends in single-drug substitution over time in ART. However, our findings should be considered in light of the study limitations. First, this study represents patients from only one government ART site and may, therefore, not be generalizable to other clinics. Second, due to the lack of documentation of reasons for single-drug substitution for 42.6% of events amongst patients on a stavudine-based regimen, roughly 75% of events amongst patients on a tenofovir- or zidovudine-based regimen and over 55% of events amongst patients on nevirapine- or efavirenz-based regimens, we are likely underestimating the frequency and type of side effects due to less than perfect surveillance. Third, multiple imputation helps make it possible to handle missing data routinely and improve the validity of research. However, the procedure requires the user to model the distribution of each variable with missing values in terms of the observed data [24]. The validity of results from multiple imputation depends on such modelling being done carefully and is based on the assumption that our data are missing at random. Deviations from this could have led to unpredictable biases in our parameter estimates.

## Conclusions

The decline in single-drug substitutions in the first 12 months on ART is associated with the use of tenofovir in first-line regimens in our cohort, resulting in few drug toxicities in patients on treatment and improved regimen durability. Although the increased use of tenofovir could improve treatment outcomes in resource-limited settings, further research is necessary to determine if a lower dose of stavudine may be a valid option in countries where reduction in the use of stavudine will be progressive, including those with limited access to drug alternatives and/or limited laboratory capabilities, and as a back-up option in the presence of treatment-limiting toxicity due to zidovudine or tenofovir.
